# Divide and Be Conquered—Cell Cycle Reactivation in Arbuscular Mycorrhizal Symbiosis

**DOI:** 10.3389/fpls.2021.753265

**Published:** 2021-10-25

**Authors:** Giulia Russo, Andrea Genre

**Affiliations:** ^1^Department of Agricultural, Forest, and Food Sciences, University of Turin, Turin, Italy; ^2^Department of Life Science and Systems Biology, University of Turin, Turin, Italy

**Keywords:** arbuscular mycorrhizal, cell cycle, symbiosis, cell division, endoreduplication

## Introduction

Arbuscular mycorrhizas (AM) are widespread symbiotic associations between 78% of vascular plant species globally (Tedersoo et al., 2020) and soil borne Glomeromycotina fungi (Spatafora et al., 2016). Analogous associations with fungi appeared over 400 million years ago (Strullu-Derrien et al., 2014, 2018) and are thought to have played a major role in the transition of plants from aquatic to terrestrial environments (Wang et al., 2010). Indeed, extant AM fungi supply their host plants with water and mineral nutrients, resources that are as indispensable for them as they were for their earliest ancestors (Feijen et al., 2018).

AM development is a conserved process across extant host species with limited morphological variations in the symbiotic structures (Choi et al., 2018). Following an exchange of chemical signals (Zipfel and Oldroyd, 2017) root colonization starts with the formation of a hyphopodium on the root surface (Kobae et al., 2018). Fungal entry in epidermal cells is anticipated by the assembly of the prepenetration apparatus (PPA), a broad, nucleus-associated cytoplasmic bridge. Here, exocytic and endocytic processes (Genre et al., 2005, 2008, 2012; Russo et al., 2019b) contribute to build a novel cell compartment, the symbiotic interface, hosting hyphae within an invagination of the plant cell membrane and a layer of unstructured cell wall components (Balestrini et al., 1996; Parniske, 2008; Balestrini and Bonfante, 2014). Such symbiotic interfaces accommodate all intracellular hyphae as they develop toward the inner cortex, where their repeated branching originates arbuscules: the distinctive structures of this symbiosis, where mineral nutrients and water are transferred to the plant across the extensive periarbuscular interface (Luginbuehl and Oldroyd, 2017), in exchange for sugars (Roth and Paszkowski, 2017) and lipids (Keymer and Gutjahr, 2018).

The study of fungal accommodation has revealed that host cell rearrangement, calcium-mediated signals and major changes in gene expression extend to neighboring, uncolonized cells (Genre et al., 2008; Pumplin and Harrison, 2009; Gaude et al., 2012; Sieberer et al., 2012), indicating that signaling processes anticipate fungal development within the root tissues. In this context, we have recently shown that such prepenetration responses include cell cycle reactivation in cortical cells, with anticlinal cell divisions and recursive endoreduplication anticipating fungal colonization (Carotenuto et al., 2019a,b; Russo et al., 2019a,b). We here propose a model depicting the recruitment of cell cycle processes as a strategy for arbuscule accommodation, speculating on its conservation in other, more recent, biotrophic interactions.

## Cell Division and Endoreduplication Anticipate Arbuscule Accommodation

Several studies had reported an increase in ploidy in mycorrhizal roots of different angiosperms (Berta et al., 2000; Fusconi et al., 2005; Bainard et al., 2011). Siciliano et al. (2007) reported that histone H2B1 gene was induced in root segments of *Medicago truncatula* on which hyphopodium formation had occurred, supporting the hypothesis that cell divisions determinants are co-opted by the plant cell in preparation of interface compartment construction. More recently, combined microscopy, flow cytometry and gene expression studies have revealed that the activation of cell division- related processes occurs since the early steps of AM development, often at a distance from the colonizing hyphae (Carotenuto et al., 2019a,b; Russo et al., 2019a,b). Firstly, the presence of sparse couples of “split cells” in the inner root cortex was consistently observed in both young and fully developed colonization units from diverse plants. Such split cells were half the length of the surrounding parenchymal cells, suggesting the occurrence of cell division after tissue differentiation. This was confirmed by the direct observation of dividing cells as early as 48 h post-hyphopodium formation in *Daucus carota* expressing a GFP fusion with TPLATE (Russo et al., 2019a), an adaptin-related protein that accumulates on the cell plate membrane and plasmalemma at the cortical division zone (Van Damme et al., 2006).

Furthermore, taking advantage of the correlation between flow cytometry data and detailed nuclear size measurements through confocal imaging, the precise localization of inner cortical cells with different levels of increased ploidy in the AM colonized areas was achieved (Carotenuto et al., 2019a). This revealed the diffuse occurrence of endoreduplication events—i.e., DNA duplication in the absence of cell division (Barow, 2006)—throughout AM development (Carotenuto et al., 2019b), as supported by the upregulation of several key endocycle and S-phase marker genes (Carotenuto et al., 2019a), such as negative regulators of G2-M-specific cyclins *MtAPC/C subunit 2* (Tarayre et al., 2004) and *MtCCS52A* (Cebolla et al., 1999), and markers of DNA replication during the S phase, such as two subunits of DNA Topoisomerase VI, *MtVAG1*, and *MtTOPO-VI B* (Bergerat et al., 1994, 1997), and the histone *MtHist-H4* (Lepetit et al., 1992).

In addition, uncolonized split cells often displayed lower ploidy than their neighboring undivided cells, suggesting that cell division and endoreduplication combine to generate the resulting mixed population of cells with diverse ploidy levels (Carotenuto et al., 2019b).

These observations outlined a previously unpredicted scenario of cell cycle reactivation in response to AM colonization. Attempting to explain the origin and role of these conserved and histologically localized responses, a few additional considerations should be discussed.

Firstly, cell divisions in the inner cortex have been observed when intraradical hyphae were limited to epidermal and outer cortical layers but not in later stages; by contrast, as demonstrated by combined flow cytometry and microscopy data, recursive endoreduplication cycles appear to be active for a longer period of time, with arbusculated and neighboring cells reaching levels of 128C ploidy, corresponding to up to 5 cycles of endoreduplication (Carotenuto et al., 2019a). In more detail, confocal imaging revealed that the increase in nuclear size—a hallmark of endoreduplication—surged at the front of fungal expansion and reached the highest peaks in the central area of infection units, suggesting the existence of a proportion between ploidy and the abundance (or age) of intraradical fungal structures. Importantly, Carotenuto et al. (2019b) also observed that the couples of split cortical cells derived from cell division often displayed different nuclear sizes, with larger nuclei in cells that were closer to the fungus or hosting an older arbuscule. This strongly suggests that cell division takes place before endoreduplication, or at least that endoreduplication can proceed after cell division.

Secondly, the observation of both cell division and ploidy increase at a distance from arbuscules or colonizing hyphae suggests the existence of a yet unidentified signaling process reactivating the cell cycle before fungal arrival.

In addition, the concentration of both ectopic cell divisions and endocycle events to the inner cortex envisages a remarkable correlation with the accommodation of arbuscules, which normally develop in the same cell layer. Cell proliferation, with its limited occurrence, appears to have a secondary role, if any, in the generation of additional space for arbuscule accommodation. By contrast, the sparse cell divisions observed in AM colonized areas might relate to the developmental fate of cortical cells. In the roots of most plants, in fact, cortical cell differentiation is determined with an endocycle that doubles their DNA content from 2C to 4C (Cebolla et al., 1999; Edgar et al., 2014) with a consequent size increase (Robinson et al., 2018). In line with that, *in situ* studies of cell ploidy in uncolonized roots of *M. truncatula* (Carotenuto et al., 2019a) revealed that most cortical cells had 4C nuclei, while a few of them displayed 8C and 16C ploidy levels. Even if experimentally challenging, it would be very interesting to investigate if there is a relationship between initial cell ploidy and the occurrence of ectopic cell division in early AM interaction.

Besides tissue differentiation, endoreduplication is also common in plant interactions with diverse microbes: replicating DNA produces multiple copies of each gene, intensifying cell responsiveness to microbial signals. Examples are numerous, from pathogens and parasites (de Almeida Engler and Gheysen, 2013; Chandran and Wildermuth, 2016; Wildermuth et al., 2017) to symbionts (Suzaki et al., 2014; Lace and Ott, 2018). Furthermore, endoreduplication-related cell enlargement is typically associated with the accommodation of several microbes, and specifically to arbuscules in AM (Balestrini and Bonfante, 2014; Heck et al., 2016).

The requirement of a specific rearrangement in the host cell organization for arbuscule accommodation is apparent from a simple observation of the structural and functional complexity of the periarbuscular interface (Luginbuehl and Oldroyd, 2017; Ivanov et al., 2019; Roth et al., 2019), compared to the tunnel-like interface hosting linear hyphae in outer root tissues. In fact, while epidermal and outer cortical PPAs are structured as roughly linear cytoplasmic bridges across the vacuole, the PPAs that generate periarbuscular interfaces are much more complex and extensive, appearing as large accumulations of cytoplasm that extend from the hyphal penetration site and occupy most of the host cell central volume (Genre et al., 2008). Such a massive, centripetally-oriented exocytic event has striking ultrastructural and molecular similarities with the assembly of the cell plate on the cell equatorial plane at the end of mitosis, and indeed an evolutionary correlation between symbiotic interface biogenesis and cell plate deposition has been envisaged in both AM (Russo et al., 2019b) and N-fixing nodulation (Downie, 2014). In support of this hypothesis, *in vivo* imaging of GFP-TPLATE fusions revealed a strong accumulation of TPLATE at sites of PPA assembly and at sites of cell-to-cell hyphal passage, where the perifungal membrane fuses with the plasmalemma, in striking analogy with cell plate fusion with the cell membrane at the end of mitosis (Russo et al., 2019b).

If the recruitment of cell division processes to assemble the extensive periarbuscular interface now appears more convincing, developmental restraints could contribute to explain why sparse cell division and diffuse endoreduplication are limited to the cortex. Dong et al. (2021) have recently highlighted that a SHR-SCR module (known to regulate cortex/endodermis initial cell division in the root meristem) maintains its activity and is required for cell cycle reactivation in legume inner cortex during nodule organogenesis (Suzaki et al., 2014; Xiao et al., 2014). Even if analogous studies in rice (which does not form root nodules, but hosts AM fungi) did not confirm SHR-SCR expression in cortical cells, it is reasonable to speculate that analogous mechanisms involving meristematic transcription factors maintain a disposition to reactivate the cell cycle in inner cortical cells. This peculiarity has been related to the evolution of root branching (Xiao et al., 2019), but appears to have later been co-opted in several plant interactions, from N-fixing symbioses (Dong et al., 2021) to nematode parasitism (de Almeida Engler and Gheysen, 2013), where both cell division and endoreduplication are required for microbe accommodation. While anyway such processes involve the formation of new organs (i.e. lateral roots, N-fixing nodules or nematode-hosting cysts), their occurrence in AM, where organogenesis is absent, appears puzzling; even more so if we consider that AM symbiosis appeared in land plants before the evolution of true roots (Strullu-Derrien et al., 2014, 2018).

## Conclusions

**Figure 1:**
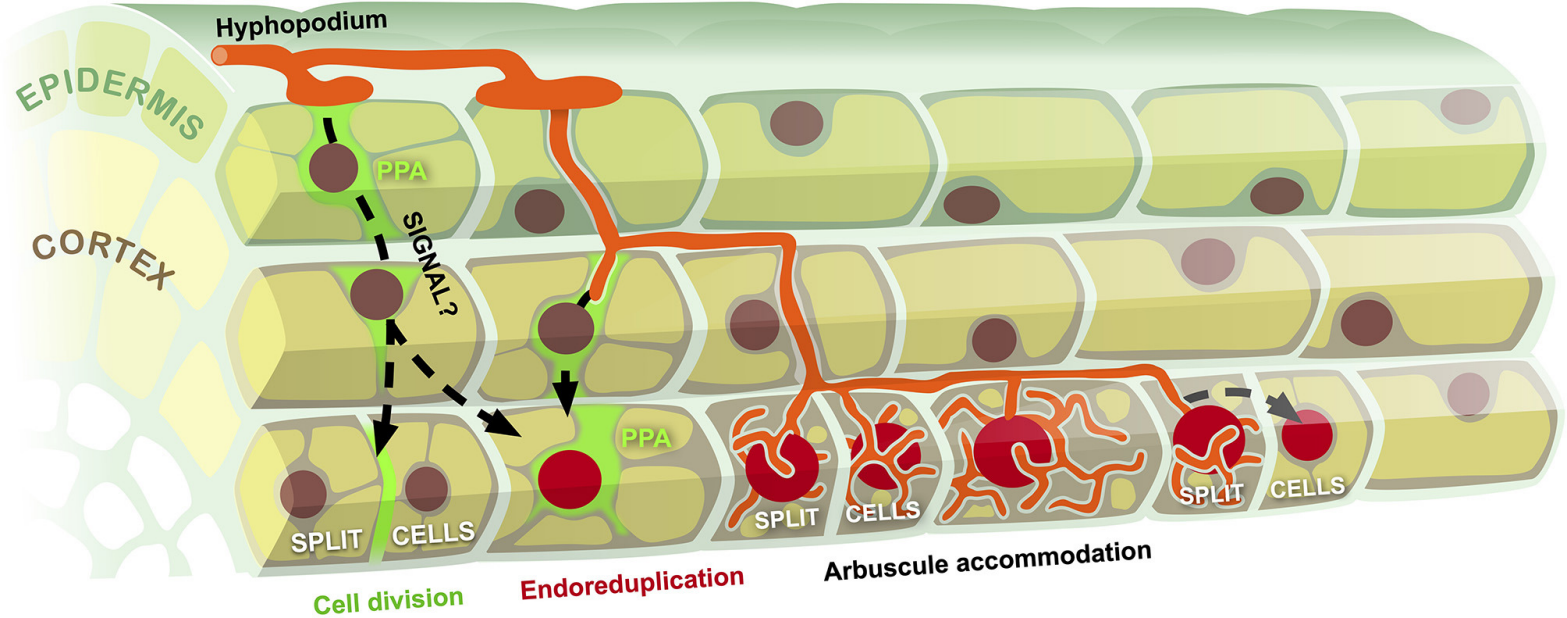
Model of cell cycle reactivation during fungal accommodation in AM. Hyphal colonization of the root epidermis associated with prepenetration apparatus assembly (PPA) triggers a so far uncharacterized signaling process (black dashed arrows) targeting inner cortical cells. This causes the reactivation of cell cycle processes, leading to occasional cell divisions (split cells) and diffuse events of endoreduplication (large red nuclei). Progressive intraradical development of the symbiotic fungus reiterates the stimulation of cell cycle activation, leading to multiple rounds of endoreduplication in advance of arbuscule accommodation and to the expansion of the endoreduplication zone at the front of the developing colonization unit.

By discussing the developmental and evolutionary context where cell cycle processes interweave with AM symbiosis, a scenario emerges (Figure 1) where the perception of AM fungal colonization in outer root tissues triggers a so-far unknown intraradical signaling process activating cell cycle-related processes ahead of the penetrating intraradical hyphae. Inner cortical cells may deploy two downstream responses: a few of them (possibly depending on their ploidy) complete mitosis, splitting in two smaller cells, as cell elongation is very limited in a mature tissue (Russo et al., 2019a,b); the remaining majority of inner cortical cells enter the endocycle, duplicating their DNA content up to several times, continuously stimulated by the approaching fungal symbiont—in fact endoreduplication also extends to those cells that had divided earlier (Carotenuto et al., 2019a,b). Such a model implies that host cells largely anticipate and direct fungal colonization, in line with previous propositions that the plant holds substantial control over symbiosis development (Parniske, 2008).

We currently have no information on how the earliest land plants acquired the ability to host a symbiotic fungus inside their cells. One can speculate that initial surface interactions provided an advantageous exchange of nutrients, pressing toward more intimate contacts, such as the penetration of fungal hyphae between the plant cells and eventually inside their lumen. In this context, creating *de novo* a fully functional symbiotic interface—as in modern plants—appears unrealistic. By contrast, stimulating cell divisions in differentiated organs could have been a more amenable strategy to generate both crack openings in the surface tissues (an entry route that is conserved in many extant plant-microbe interactions; Ibáñez et al., 2017) and irregular intercellular spaces in the inner ones, producing a protected niche for the fungus. The subsequent re-routing of cell plate formation toward the creation of a more efficient symbiotic interface appears achievable, especially in the light of the current findings, and the observation of split cells in some of the earliest fossils of AM hosts indicates that this is indeed an ancient response associated with fungal accommodation (Strullu-Derrien et al., 2018).

## Author Contributions

Both authors made a substantial, direct and intellectual contribution to the work, and approved it for publication.

## Conflict of Interest

The authors declare that the research was conducted in the absence of any commercial or financial relationships that could be construed as a potential conflict of interest.

## Publisher's Note

All claims expressed in this article are solely those of the authors and do not necessarily represent those of their affiliated organizations, or those of the publisher, the editors and the reviewers. Any product that may be evaluated in this article, or claim that may be made by its manufacturer, is not guaranteed or endorsed by the publisher.

## References

[B1] Bainard L. D. Bainard J. D. Newmaster S. G. Klironomos J. N. (2011). Mycorrhizal symbiosis stimulates endoreduplication in angiosperms. Plant Cell Environ. 34, 1577–1585. 10.1111/j.1365-3040.2011.02354.x 21707648

[B2] Balestrini R. Bonfante P. (2014). Cell wall remodeling in mycorrhizal symbiosis: a way towards biotrophism. Front. Plant Sci. 5:237. 10.3389/fpls.2014.00237 24926297 PMC4044974

[B3] Balestrini R. Hahn M. G. Faccio A. Mendgen K. Bonfante P. (1996). Differential localization of carbohydrate epitopes in plant cell walls in the presence and absence of arbuscular mycorrhizal fungi. Plant Physiol. 111, 203–213. 10.1104/pp.111.1.203 12226286 PMC157827

[B4] Barow M. (2006). Endopolyploidy in seed plants. BioEssays. 28, 271–281. 10.1002/bies.20371 16479577

[B5] Bergerat A. de Massy B. Gadelle D. Varoutas P. C. Nicolas A. Forterre P. (1997). An atypical607 topoisomerase II from Archaea with implications for meiotic recombination. Nature 386, 414–417. 9121560 10.1038/386414a0

[B6] Bergerat A. Gadelle D. Forterre P. (1994). Purification of a DNA topoisomerase II from the hyperthermophilic archaeon *Sulfolobus shibatae*. A thermostable enzyme with both bacterial and eucaryal features. J. Biol. Chem.. 269, 27663–27669. 10.1016/S0021-9258(18)47037-8 7961685

[B7] Berta G. Fusconi A. Sampo S. Lingua G. Perticone S. Repetto O. (2000). Polyploidy in tomato roots as affected by arbuscular mycorrhizal colonization. Plant Soil. 226, 37–44. 10.1007/978-94-017-2858-4_8

[B8] Carotenuto G. Sciascia I. Oddi L. Volpe V. Genre A. (2019b). Size matters: three methods for estimating nuclear size in mycorrhizal roots of *Medicago truncatula* by image analysis. BMC Plant Biol. 19:180. 10.1186/s12870-019-1791-1 31054574 PMC6500585

[B9] Carotenuto G. Volpe V. Russo G. Politi M. Sciascia I. de Almeida-Engler J.. (2019a). Local endoreduplication as a feature of intracellular fungal accommodation in arbuscular mycorrhizas. New Phytol. 223, 30–446. 10.1111/nph.15763 30811604

[B10] Cebolla A. Vinardell J. M. Kiss E. Oláh B. Roudier F. Kondorosi A.. (1999). The mitotic inhibitor ccs52 is required for endoreduplication and ploidy-dependent cell enlargement in plants. EMBO J. 18, 4476–4484. 10.1093/emboj/18.16.4476 10449413 PMC1171522

[B11] Chandran D. Wildermuth M. C. (2016). Modulation of host endocycle during plant-biotroph interactions. Enzymes. 40, 65–103. 10.1016/bs.enz.2016.09.001 27776783

[B12] Choi J. Summers W. Paszkowski U. (2018). Mechanisms underlying establishment of arbuscular mycorrhizal symbioses. Annu. Rev. Phytopathol. 56, 135–160. 10.1146/annurev-phyto-080516-035521 29856935

[B13] de Almeida Engler J. Gheysen G. (2013). Nematode-induced endoreduplication in plant host cells: why and how? Mol. Plant. Microbe. Interact. 26, 17–24. 10.1094/MPMI-05-12-0128-CR 23194340

[B14] Dong W. Zhu Y. Chang H. Wang C. Yang J. Shi J.. (2021). An SHR-SCR module specifies legume cortical cell fate to enable nodulation. Nature. 589, 586–590. 10.1038/s41586-020-3016-z 33299183

[B15] Downie J. A. (2014). Legume nodulation. Curr. Biol. 24, 184–190. 10.1016/j.cub.2014.01.028 24602880

[B16] Edgar B. Zielke N. Gutierrez C. (2014). Endocycles: a recurrent evolutionary innovation for post-mitotic cell growth. Nat. Rev. Mol. Cell. Biol. 15, 197–210. 10.1038/nrm3756 24556841

[B17] Feijen F. A. A. Vos R. A. Nuytinck J. Merckx V. S. F. T. (2018). Evolutionary dynamics of mycorrhizal symbiosis in land plant diversification. Sci. Rep. 8:10698. 10.1038/s41598-018-28920-x 30013185 PMC6048063

[B18] Fusconi A. Lingua G. Trotta A. Berta G. (2005). Effects of arbuscular mycorrhizal colonization and phosphorus application on nuclear ploidy in Allium porrum plants. Mycorrhiza. 15, 313–321. 10.1007/s00572-004-0338-x 15565274

[B19] Gaude N. Bortfeld S. Duensing N. Lohse M. Krajinski F. (2012). Arbuscule containing and non-colonized cortical cells of mycorrhizal roots undergo extensive and specific reprogramming during arbuscular mycorrhizal development. Plant J. 69, 510–528. 10.1111/j.1365-313X.2011.04810.x 21978245

[B20] Genre A. Chabaud M. Faccio A. Barker D. G. Bonfante P. (2008). Prepenetration apparatus assembly precedes and predicts the colonization patterns of arbuscular mycorrhizal fungi within the root cortex of both *Medicago truncatula* and *Daucus carota*. Plant Cell. 20, 1407–1420. 10.1105/tpc.108.059014 18515499 PMC2438458

[B21] Genre A. Chabaud M. Timmers T. Bonfante P. Barker D. G. (2005). Arbuscular mycorrhizal fungi elicit a novel intracellular apparatus in *Medicago truncatula* root epidermal cells before infection. Plant Cell. 17, 3489–3499. 10.1105/tpc.105.035410 16284314 PMC1315383

[B22] Genre A. Ivanov S. Fendrych M. Faccio A. Zársky V. Bisseling T.. (2012). Multiple exocytotic markers accumulate at the sites of perifungal membrane biogenesis in arbuscular mycorrhizas. Plant Cell Physiol. 53, 244–255. 10.1093/pcp/pcr170 22138099

[B23] Heck C. Kuhn H. Heidt S. Walter S. Rieger N. Requena N. (2016). Symbiotic fungi control plant root cortex development through the novel GRAS transcription factor MIG1. Curr. Biol. 26, 2770–2778. 10.1016/j.cub.2016.07.059 27641773

[B24] Ibáñez F. Wall L. Fabra A. (2017). Starting points in plant-bacteria nitrogen-fixing symbioses: intercellular invasion of the roots. J. Exp. Bot. 68, 1905–1918. 10.1093/jxb/erw387 27756807

[B25] Ivanov S. Austin J. Berg R. H. Harrison M. J. (2019). Extensive membrane systems at the host-arbuscular mycorrhizal fungus interface. Nat. Plants. 5, 194–203. 10.1038/s41477-019-0364-5 30737512

[B26] Keymer A. Gutjahr C. (2018). Cross-kingdom lipid transfer in arbuscular mycorrhiza symbiosis and beyond. Curr. Opin. Plant Biol. 44, 137–144. 10.1016/j.pbi.2018.04.005 29729528

[B27] Kobae Y. Kameoka H. Sugimura Y. Saito K. Ohtomo R. Fujiwara T.. (2018). Strigolactone biosynthesis genes of rice is required for the punctual entry of arbuscular mycorrhizal fungi into the roots. Plant Cell Physiol. 59, 544–553. 10.1093/pcp/pcy001 29325120

[B28] Lace B. Ott T. (2018). Commonalities and differences in controlling multipartite intracellular infections of legume roots by symbiotic microbes. Plant Cell Physiol. 59, 666–677. 10.1093/pcp/pcy043 29474692

[B29] Lepetit M. Ehling M. Chaubet N. Gigot C. (1992). A plant histone gene promoter can direct both replication-dependent and -independent gene expression in transgenic plants. Mol. Gen. Genet. 231, 276–285. 10.1007/BF00279801 1736097

[B30] Luginbuehl L. H. Oldroyd G. E. D. (2017). Understanding the arbuscule at the heart of endomycorrhizal symbioses in plants. Curr. Biol. 27, R952–R963. 10.1016/j.cub.2017.06.042 28898668

[B31] Parniske M. (2008). Arbuscular mycorrhiza: the mother of plant root endosymbioses. Nat. Rev. Microbiol. 6, 763–775. 10.1038/nrmicro1987 18794914

[B32] Pumplin N. Harrison M. J. (2009). Live-cell imaging reveals periarbuscular membrane domains and organelle location in *Medicago truncatula* roots during arbuscular mycorrhizal symbiosis. Plant Physiol. 151, 809–819. 10.1104/pp.109.141879 19692536 PMC2754618

[B33] Robinson D. O. Coate J. E. Singh A. Hong L. Bush M. Doyle J. J.. (2018). Ploidy and size at multiple scales in the arabidopsis sepal. Plant Cell. 30, 2308–2329. 10.1105/tpc.18.00344 30143539 PMC6241276

[B34] Roth R. Hillmer S. Funaya C. Chiapello M. Schumacher K. Lo Presti L.. (2019). Arbuscular cell invasion coincides with extracellular vesicles and membrane tubules. Nat. Plants. 5, 204–211. 10.1038/s41477-019-0365-4 30737514

[B35] Roth R. Paszkowski U. (2017). Plant carbon nourishment of arbuscular mycorrhizal fungi. Curr. Opin. Plant Biol. 39, 50–56 10.1016/j.pbi.2017.05.008 28601651

[B36] Russo G. Carotenuto G. Fiorilli V. Volpe V. Chiapello M. Van Damme D.. (2019a). Ectopic activation of cortical cell division during the accommodation of arbuscular mycorrhizal fungi. New Phytol. 221, 1036–1048. 10.1111/nph.15398 30152051

[B37] Russo G. Carotenuto G. Fiorilli V. Volpe V. Faccio A. Bonfante P.. (2019b). TPLATE recruitment reveals endocytic dynamics at sites of symbiotic interface assembly in arbuscular mycorrhizal interactions. Front. Plant Sci. 10:1628. 10.3389/fpls.2019.01628 31921269 PMC6934022

[B38] Siciliano V. Genre A. Balestrini R. Cappellazzo G. deWit P. J. Bonfante P. (2007). Transcriptome analysis of arbuscular mycorrhizal roots during development of the prepenetration apparatus. Plant Physiol. 144, 1455–1466. 10.1104/pp.107.097980 17468219 PMC1914140

[B39] Sieberer B. J. Chabaud M. Fournier J. Timmers A. C. Barker D. G. (2012). A switch in Ca2+ spiking signature is concomitant with endosymbiotic microbe entry into cortical root cells of *Medicago truncatula*. Plant J. 69, 822–830. 10.1111/j.1365-313X.2011.04834.x 22035171

[B40] Spatafora J. W. Chang Y. Benny G. L. Lazarus K. Smith M. E. Berbee M. L.. (2016). A phylum-level phylogenetic classification of zygomycete fungi based on genome-scale data. Mycologia. 108, 1028–1046. 10.3852/16-042 27738200 PMC6078412

[B41] Strullu-Derrien C. Kenrick P. Pressel S. Duckett J. G. Rioult J. P. Strullu D. G. (2014). Fungal associations in Horneophyton ligneri from the Rhynie Chert (c. 407 million year old) closely resemble those in extant lower land plants: novel insights into ancestral plant-fungus symbioses. New Phytol. 203, 964–79. 10.1111/nph.12805 24750009

[B42] Strullu-Derrien C. Selosse M. A. Kenrick P. Martin F. M. (2018). The origin and evolution of mycorrhizal symbioses: from palaeomycology to phylogenomics. New Phytol. 220, 1012–1030. 10.1111/nph.15076 29573278

[B43] Suzaki T. Ito M. Yoro E. Sato S. Hirakawa H. Takeda N.. (2014). Endoreduplication-mediated initiation of symbiotic organ development in *Lotus japonicus*. Development. 141, 2441–2445. 10.1242/dev.107946 24850853

[B44] Tarayre S. Vinardell J. M. Cebolla A. Kondorosi A. Kondorosi E. (2004). Two classes of the cdh1-type activators of the anaphase-promoting complex in plants: novel functional domains and distinct regulation. Plant Cell. 16, 422–434. 10.1105/tpc.018952 14742878 PMC341914

[B45] Tedersoo L. Bahram M. Zobel M. (2020). How mycorrhizal associations drive plant population and community biology. Science. 367, 867–876. 10.1126/science.aba1223 32079744

[B46] Van Damme D. Coutuer S. De Rycke R. Bouget F. Y. Inzé D. Geelen D. (2006). Somatic cytokinesis and pollen maturation in Arabidopsis depend on TPLATE, which has domains similar to coat proteins. Plant Cell. 18, 3502–3518. 10.1105/tpc.106.040923 17189342 PMC1785392

[B47] Wang B. Yeun L. H. Xue J. Y. Liu Y. Ané J. M. Qiu Y. L. (2010). Presence of three mycorrhizal genes in the common ancestor of land plants suggests a key role of mycorrhizas in the colonization of land by plants. New Phytol. 186, 514–525. 10.1111/j.1469-8137.2009.03137.x 20059702

[B48] Wildermuth M. C. Steinwand M. A. McRae A. G. Jaenisch J. Chandran D. (2017). Adapted biotroph manipulation of plant cell ploidy. Annu. Rev. Phytopathol. 55, 537–564. 10.1146/annurev-phyto-080516-035458 28617655

[B49] Xiao T. T. Schilderink S. Moling S. Deinum E. E. Kondorosi E. Franssen H.. (2014). Fate map of *Medicago truncatula* root nodules. Development. 141, 3517–3528. 10.1242/dev.110775 25183870

[B50] Xiao T. T. van Velzen R. Kulikova O. Franken C. Bisseling T. (2019). Lateral root formation involving cell division in both pericycle, cortex and endodermis is a common and ancestral trait in seed plants. Development. 146:dev182592. 10.1242/dev.182592 31591087

[B51] Zipfel C. Oldroyd G. E. (2017). Plant signalling in symbiosis and immunity. Nature. 543, 328–336. 10.1038/nature22009 28300100

